# Popular culture and genetics; friend, foe or something more complex?

**DOI:** 10.1016/j.ejmg.2018.12.005

**Published:** 2019-05

**Authors:** Jonathan Roberts, Louise Archer, Jennifer DeWitt, Anna Middleton

**Affiliations:** a Wellcome Genome Campus, Society and Ethics Research and Addenbrooke's Hospital, United Kingdom; b Wellcome Genome Campus Society and Ethics Research and Faculty of Education, University of Cambridge, United Kingdom; c University College London, Institute of Education, United Kingdom

## Abstract

While many people enjoy popular culture, these transactional experiences may not translate into formal or academic learning about a subject. In education and science communication settings popular culture is often presented as a source of inaccurate information about science. Different publics are often positioned as, at best, undiscriminating consumers and at worst victims of distorted scientific information. We explore how people use their own knowledge and interests to engage with genetics. Here, data from family interviews are used to illustrate how participants draw on popular culture as a resource to engage with and articulate their beliefs about genetics. Using qualitative data from family interviews we describe two perspectives: first, popular culture represents a source of narratives and metaphors used for rhetorical purposes. Second participants used fictional narratives in more depth - as sense-making devices - allowing people to explore the moral and ethical implications of genetics. We argue that by utilising patients’ interests – such as popular culture – we can potentially enrich communication in a genetic counselling context.

## 1 Introduction

Genomics is predicted to impact the lives of people as patients, consumers and citizens in a myriad of different ways (Roberts and Middleton, 2018). Genomic technology is now being utilised in more settings across society than ever before, including medicine, population health screening, recreational consumerism (ancestry testing, nutritional testing), through to policing and crime prevention. Signifying the importance placed on genomics, the most recent annual report of the Chief Medical Officer of the United Kingdom (2017) was entitled “Generation Genome” and stated:

Genomics is not tomorrow. It's here today. I believe genomic services should be available to more patients, whilst being a cost-effective service in the NHS. This is exciting science with the potential for fantastic improvements in prevention, health protection and patient outcomes. Now we need to welcome the genomic era and deliver the genomic dream! (Davies, 2013 p.1 p.1)

As genomics increases in prevalence, patients, consumers and citizens are increasingly being asked to make meaning of genetic information. As such, attention has been paid to the way publics' understand and relate to genetic information. In addition to formal educational activities, scholarship has explored the impact of popular culture on how people perceive ‘genetics’ as a discipline. This paper presents findings from a study that explored the how people draw on their own knowledge and interests to engage with genetics. Here, data from family interviews are used to illustrate how participants are able to draw on popular culture as a resource to engage with and articulate their beliefs about genetics.

## 2 Science, genetics and popular culture

Scholars have argued that popular culture and fictional representations of science offer a source of information that can be as important to the public as the ‘real’ science that is known (Nelkin and Lindee, 1996; Turney and Haynes, 1998). However, science as presented in the media has been criticised by scientists and educators as misleading, with some arguing that it provides a shallow and inaccurate depiction of genetics (Michael and Carter, 2001). Such concerns are reflected in work which highlights the deterministic implications of simplistic narratives of genetics (Lippman 1993).

Media representations of genetics are regularly criticised by scientists and policy makers as an exaggeration or fear mongering (Vackimes, 2010). This is linked to a deficit view that regards public knowledge as shallow and reactionary (Haran & O'Riordan, 2017). With genetic technology prominent in horror, science fiction and dystopian fiction (Haran et al., 2007) policy makers and scholars have expressed concerns that fictional representations, in particular, stoke irrational fears regarding genetic technologies (Hughes and Kitzinger, 2008). Huxford (2000) for example, in his analysis of cloning in the media, writes that that there are many “anti-science themes intrinsic to science fiction” and that the undercurrent of popular science fiction is “the fear of science itself” (Huxford, 2000, p.193). Anxiety about the representations of genetics is also seen in responses to how news reports and documentaries utilise dramatic images, often drawn from fiction, in science communication. A prominent example being the use of “Frankenstein foods” to describe genetically modified food (Cook et al., 2004; Hughes and Kitzinger, 2008).

Related to concerns about the media stoking ‘irrational’ fears about science is the effect popular culture has on science literacy. Here researchers and policy makers have raised concerns that exposure to science in popular culture leads to inaccurate beliefs (Marsh et al., 2003). As an example in 2000 the US National Science Foundation suggested that “fictional media have corroded the public's critical thinking skills and have hindered scientific literacy” (Kirby, 2000, p.262) and in 2010 the President of the Federation of Australian Scientific and Technological Societies stated that Australians' “worrying science literacy levels demonstrates that students have perhaps been learning about science through Jurassic Park instead of through the education system” (quoted in Orthia et al., 2012, p.150). Science in film has come under particular criticism. Sophia Vackimes, from the Max Plank Institute for the History of Science, provides the following assessment of thirty years of genetics in film:

It is a pity that as scientific information has become more and more complex cinema has become more and more a medium that emphasizes a-historical, unscientific, and culturally contradictory positions to exploit fears while creating a climate of disinformation (Vackimes, 2010)

In a response to these concerns many articles, books and websites have been developed, attempting to utilise the reach of popular culture to teach the ‘good’ science, often by critiquing them through errors (Bixler, 2007; Dubeck et al., 2006; Rose, 2008; Smith, 2009).

Finally, researchers have raised concerns that popular culture is a widespread source of essentialist imagery and subsequently is a cause of genetic determinism. An influential book in this regard was *The DNA Mystique* by Nelkin and Lindee (1996). This work argued that human behaviour and social issues are being reshaped and recast through genetic imagery. They contend that genes and DNA have become a “cultural icons” with their representations in popular culture in particular encouraging genetic determinism. In a similar way Heine, Dar-Nimrod et al. (2016) argue that commonplace exposure to essentialist imagery in film and in the press contributes to the implicit endorsement of “genes as the essence of personhood” (p.144). These authors believe that this is far from a trivial matter arguing that genetic determinism leads to outcomes such as racism, sexism, pessimism in the face of illness and support for eugenics (Hein, Dar-Nimrod et al., 2017).

Much of the research that raises concerns about representations of science in the media has focused on content analysis, exploring how the science is represented in specific texts. Scholars have noted this research has often marginalised everyday audiences, or the ‘lay public’ from discussion (Hughes and Kitzinger, 2008). However, in recent years there have been a growing number of research studies demonstrating that audiences deal with texts actively, selectively and critically, using them as a discursive resource rather than absorbing the content in a linear manner (Condit, 1999). Michael and Carter (2001), for example, researched student's understanding of human genetics demonstrating how their participants dealt critically with a variety of sources including magazines, fictional genres and scientific textbooks. In this study the boundary created by students between fact and fiction was fluid as students were able to utilise media-derived knowledge in critical ways. As Michael and Carter put it, “the students found facts in fictions and fictions in facts” (Michael and Carter, 2001 p28). In a similar way Bates (2005) argues that through “public culture” people are able to make sense of science and the scientized culture in which they live. Specifically, he found that participants in his study were able to utilise references from popular culture with an awareness that these were fictional texts and should be treated as such. He concludes that people do not process the science depicted in media in a linear fashion, absorbing it wholesale. Rather, people negotiate its meanings within the broader context of their lives and their experiences, with conscious awareness of genre and medium. The work of Orthia et al. (2012) - which explored participants' responses to science in an episode of *The Simpsons* (popular American TV cartoon)– came to similar conclusions. They found that viewers were selective about the ways they utilised the themes that appeared in the episode they watched. Participants reflected on the science and picked out aspects of the show to articulate their own point of view.

In this study we use a series of family interviews to examine how participants were able to draw on their own knowledge to express their understanding of genetics. Mirroring some of the research outlined above, popular culture emerged as an important resource for participants when discussing genetics.

## 3 Participants and recruitment

The data presented here is taken from a larger project that explored the ways in which people are able to use their own knowledge and interests to engage with genetics. This project employed a mixed methodology with a survey (n = 1407) and 17 family interviews (n = 37). The data presented here is qualitative, taken from the family interviews.

In total 17 interviews were successfully conducted with 37 family members. Where possible, participants were interviewed with other family members. Recognising that the concept of family is increasingly fluid (Bylund et al., 2010) who constitutes as a ‘family member’ was left entirely at the discretion of the participants interviewed.

A series of semi structured interviews were conducted with each family. All interviews were audio-recorded and transcribed in full while redacting any identifying information. We position interviews as mutually constructed conversations in keeping with a social constructivist epistemology. Interviews can be intimidating or enriching affairs depending on the context. Given the ideals informing this research, we followed the interview-as-conversation approach common in some areas of qualitative research (Gomm, 2004; Kvale, 1996). However, it is also possible though that my identification as a ‘health professional and academic researcher’ could, for some, inhibit their willingness to express an opinion on aspects of genetics (see Table 1).

**Table 1:** Summary of participants.

Family members (pseudonyms)	Family group size/location	Duration of interview	Key demographics
1 Gina	1 Public Library. Saffron Walden	1h52 m	Age:52 Sex: Female Occupation: Long term unemployed Highest education qualification: School
2 Neville, Vicki	2 House. Cambridge	1h05 m	Neville: Age: 51 Sex: Male Occupation: I.T consultant. Highest education: Undergraduate Degree Vicki: Age 54 Sec: Female Occupation Retired (preciously worked in industry as a scientist) Highest Education: PhD (physics) Other: Vicki and Neville were partners.
3 Simon	1 Skype (participant lived in Leeds)	58 m	Age: 38 Sec: Male Occupation: English tutor in prisons Highest education: Undergraduate degree
4 Sarah El Jen	3 Café, Gloucester	1h12 m	Sarah: Age 38 Sex: Female Occupation: Physiotherapist Highest education: Undergraduate Interview took place with two daughters El (11) and Jen (12)
5 Lisa	1 Participant's house, Gloucester	1 h	Age:46 Sex: Female Woman 46. Occupation: Long term unemployed Highest education: Undergraduate degree
6 Riana Tina	2 Skype (participant lived Australia)	57 m	Riana Age: 22 Sec: Female Occupation: Student Highest education; completing a Master's degree in Genetics Tina (Riana's mother) Age: 51 Sex: Female Woman, 51. Highest education: Master's degree Occupation: Teacher
7 Mary David Anna Beth	4 Participants' house, Cambridge	1h5m	Mary Age 37 Sex: Female Occupation: Pilates instructor. Highest education PhD (genetics) David Age 42 Sex: Male Occupation: I.T consultant Highest education: Undergraduate degree Daughters: Children Anna (11) and Beth (8)
8 Jane, Dan, Simon and Gemma	4 Participants' house, Gloucestershire	1h22 m	Jane: Age: 38 Sex: Female Occupation: works in publishing. Highest education: Undergraduate Dan Age: 40 Sex: Male Occupation: Scientist (works for an energy company) Their children Simon (12) and Gemma (10)
9 Emma Susan	2 Café, Chester	1h45 m	Emma Age: 25 Sex: Female Occupation: Social worker Highest education: Undergraduate Susan (Emma's mother) Age: 53 Sex: Female Occupation: Secretary Highest education: School
10 Tracy Dwayne Katie Tyler	4 Participants' house, Chesterfield	2h12 m	Tracy: Age: 39 Sex: Female Occupation: Long term unemployed Highest education school (left at 16) Dwayne Age: 42 Sex: Male Occupation: Long term unemployed Highest education: School (left at 18) Children Katie (13) and Tyler (9)
11 Jessica Emily	2 House, Grimsby	58 m	Jessica: Age: 67 Sex: Female Occupation: Retired Nurse Highest education: Professional qualification Emily (Jessica's daughter) Age: 36 Occupation: Works in call centre (customer support) Highest education: School (18)
12. Abby Harry	2 Participants' house, South London	1h3m	Abby Age: 57 Sex: Female Occupation: Long Term Unemployed. Highest education: School Harry Age: 54 Sex: Male Occupation: Office worker (council) Highest education: Undergraduate degree
13. Kylie Dan	2 Participants' house, Gloucester	1h20 m	Kylie: Age: 36 Sex: Female Occupation: Full time carer (for disabled daughter) Highest education: School Dan: Age: 40 Sex: Male Occupation: Full time carer Highest education: School
14. Stephen Margaret	2 Participants' house, East London	1h13 m	Stephen: Age: 23 Sex: Male Occupation: Office worker Highest education: School (18) Margaret (Stephens mother) Age: 56 Occupation: Retired (Cleaner) Highest education: School (16)
15. Paul	Participant's house, South London	52 m	Age: 42 Sex: Male Occupation: Taxi Driver Highest education: School (18)
16 Jessica	Café; Central London	59 m	Age: 36 Sex: Female Occupation: Works in Finance Highest education: Undergraduate degree
17. Arthur Ada	House, Central London	1 h	Arthur Age: 62 Occupation: Journalist Highest education: Undergraduate degree Ada (Arthur's daughter) Age: 23 Occupation: Nursing Student Highest education: Completing undergraduate degree

## 4 Data analysis

The interviews were transcribed verbatim with the transcripts then becoming the primary component of the qualitative analysis. The interviews were analysed using thematic analysis. Broadly speaking, thematic analysis is a method used by researchers for “identifying, analysing and reporting patterns (themes) within data” (Braun & Clarke, 2006 p.79). Thematic analysis allows for a range of interpretative stances (Boyatzis, 1998). As such thematic analysis was selected as it is a form of analysis that allows a breadth of exploration whilst enabling theoretical flexibility (Braun & Clarke, 2006). Themes were generated using *a priori* assumptions (based on the research questions and the researcher's theoretical perspectives). However, elements of grounded theory are present in the sense that I was “open to the data”, allowing for themes to be generated inductively during the analytic process (Ussher and Mooney-Somers, 2000)

## 5 Findings

In this paper we outline two ways that participants were able to use popular culture. First popular culture represented source of narratives and metaphors used for rhetorical purposes. Second weI describe the ways that some participants used fictional narratives in more depth - as sense making devices - allowing people to explore the moral and ethical implications of genetics.

## 6 Popular culture: references for rhetorical purposes

Popular culture contains a repository of images, metaphors and narratives available for people to make sense of unfamiliar or new scientific concepts. These images can help people convey their emotions and attitudes about science and allow them to place issues in a shared context (Nelkin and Lindee, 1996). Gamson and Modigliani (1989) provide the concept of “interpretive packages” to describe clusters of elements such as metaphors, catchphrases, visual images, moral appeals and other symbolic devices that people use to interpret and understand the meaning of new events (Gamson and Modigliani, 1989).

We can see examples of participants utilising these *interpretive packages*, drawn from popular culture, when discussing genetics. In particular participants alluded to fictional works when articulating their opinions and beliefs about genetics. One example is a reference to Frankenstein in relation to genetic modification. Two participants made use of this reference but in different ways. Gina (a 47-year-old woman) uses this reference when expressing her (moral) distaste of genetically modified food. She says:

**Gina:** so there is a lot of debate and also the GM most of the American crops are GM genetically modified so I don't like that it’s like Frankenstein you know

This contrasts with Dwayne (man aged 38) and Tracy (woman age 36). Here Dwayne uses Frankenstein as a reference to belittle people's fear about genetic modification.

**Interviewer:** so just asking another genetics question how do you guys feel about genetically modified food does that bother you at all

**Tracy:** doesn't bother me

**Dwayne:** I think we have to do it anyway the world is not sustainable as normal food

**Tracy:** there's too many people

**Dwayne:** people are like oh it's like Frankenstein but we've got to cos there's too many people you've got to do it

These two examples demonstrate how people are able to mobilise the Frankenstein metaphor in different ways. Gina utilises the metaphor as a rhetorical device to signal the immorality and dangers of GM food. Whereas Dwayne uses it to dismiss as irrational the views of people whom, in his opinion, don't understand the importance to GM food for sustainability.

Like Gina (above) there are other examples in the data of participants using fictional references to express their concerns or fears about genetics. For example, Tina (woman aged 55) was interviewed with her daughter Riana (woman, aged 20). Tina mentions *The Boys from Brazil* she says:

**Tina:** so it was about a man cloning Hitler pretty much and it was scary and you know it could almost have been real and so those sorts of movies are interesting to me from a like oh my God it's going to happen to humanity if that actually can happen

*The Boys from Brazil (1978)* tells the story of a Nazi Hunter who discovers 94 identical genetic clones of Hitler have been created. The scientists in charge of the programme have attempted to recreate the social conditions of Hitler's youth, placing the clones with a domineering father and doting mother. Tina draws on *the Boys from Brazil* to express concern about genetics, using this as a way to succinctly articulate fears regarding human cloning.

Importantly there are many ways Boys from Brazil can be read. On the one hand the film's tag line is “if they survive … will we?” It is this deterministic imagery that creates the tension in the narrative, as the prospect of multiple clones of Hitler is only frightening if we accept that there is something evil encoded into their DNA. However, even though the film draws on these deterministic metaphors the ending remains ambiguous as to the ultimate consequences of the cloning programme. It leaves the question open as to whether the cloned Hitlers would be innately evil. So, at the same time as drawing on images of genetic determinism the film also highlights the important role of upbringing and environment in determining human characteristics.

As such, Tina's use of *The Boys from Brazil* - as a pithy reference to the dangers of genetics and cloning - could be an example of what Van Dijck (1999) calls an “imagination deficit.” Van Dijck uses this phrase to describe how she believes that, in the debate surrounding cloning, fictional works have been stripped of their nuance and limited to singular, shallow interpretations. Of the imagination deficit and the cloning debate, she says:

Relevant and interesting literary works were systematically reduced to their seemingly unequivocal or unambiguous plots, without acknowledgement of their rich, multi-interpretable and educational content. Reduction of these literary plots often reinforces conventional, flattened concepts of the human body, its identity and individuality (Van Dijck, 1999).

Above Tina says that *The Boys from Brazil* is interesting because of its plausibility. However, Tina also recognises the fictional nature of this, drawing attention to her belief that the accuracy of the science is circumspect. Kitzinger (2010) has argued that fiction may reflect concerns about science as much as they cause them. Additionally, the use of fictional references may be as much about managing competing discourses as they are about comprehension of the science (Hansen, 2006). As such, rather than viewing Tina's use of fiction as an example the imagination deficit we offer an alternative interpretation. It is possible to view this as a Tina using what she sees as a plausible and culturally resonant resource to articulate her anxiety about genetics while maintaining an awareness of the fictional nature of the text.

In their analysis of media coverage and public understanding of the Human Genome Project Durant et al. (1996) identified a polarisation between discourses of hope and discourses of fear. So far, we have predominantly seen how participants have utilised fiction in discourses of fear, drawing on images of scientists interfering with nature, playing God and creating ‘Frankenstein’ monsters.

Fiction was less common for participants to utilise in articulating hopeful visions about genetics. However, fiction was occasionally used to present optimistic visions of science and technology. Positive images of technology were generated by contrasting modern technology as looking like futuristic science fiction, noting the progress that has been made. For example, Emily (30 year old woman) says:

**Emily:** oh it's really interesting yeah cos you look at it and you think could this happen or one day could this happen

**Interviewer:** yeah

**Emily:** yeah in the future and it is fascinating like the technology in Iron man looked like impossible when it came out but now you know look more like it could happen

In the interviews, there were also examples of discourses of hope, that focused on the promise and potential of genetics. The most common sources of discourses of hope were factual genres such as news stories and documentaries. Commonly these were alluded to as sources of information about genetics and its potential to cure disease.

## 7 Popular culture: sense making and in depth use of narratives

So far we have seen how participants use popular culture and how fictional references in particular have *cultural resonance* (Gamson and Modiglani, 1989) allowing them to serve a communicative function. In the next section we describe the ways that participants use fiction to explore ethical aspects of genetics. In this sense, narratives can be thought of as tools; devices participants ‘think with.’

The first example I will discuss of fiction used as a resource in this way is *Orphan Black* a TV show that is an example of a *clone narrative*. Cloning has been seen as a fundamental threat to notions of individuality and uniqueness, and as such it is an issue that has received much attention in the media (Priest, 2001; Hopkins, 1998). According to media scholar Jimena Perez, clone narratives can engage audiences by encouraging them to reflect upon themselves. She argues that through clone fiction, audiences are encouraged to reconsider the traditional assumptions about genetics and identity (Perez, 2014).

The TV series *Orphan Black (2013–2017)* focuses on a number of women who find out that they are clones, created for mysterious reasons. In the series, the different clone's lives are shown to have taken very different paths (one is a police detective, another a scientist, another a drug addict etc). These examples explore how nature and nurture are intertwined and the ways this impacts on our perceptions of individuality and uniqueness. In addition, the show tackles the subject of commodification of genetic material when it emerges that the clones have been patented by a biotech company.

In the following interview Riana and Tina (as above) discuss how *Orphan Black.* Describing the show Riana says:

**Riana**: so, dad got me into Orphan Black a couple of years ago when I was taking a genetic course at uni and I don't know if you're familiar with it

**Interviewer**: I've seen about the first 3 or 4 episodes I think

**Riana**: so, as it goes on even though there's a lot of genetics umm there's definitely a lot about the ethics of genetics and stuff I'm really interested in so Mum didn't watch it with us

**Tina**: I didn't get into it

**Riana**: But dad and I sort of watched it every week and always sort of afterwards have sort of big discussions about the genetics in it and stuff

The extract above is an example of how fiction is used by participants as a way to facilitate a discussion about the implications of genetics within her family. Scholars have argued that fiction, especially science fiction, provides a means by which people can understand the significance of unfamiliar technologies (Appel et al., 2016). In a similar way, other media scholars have claimed that fiction can be personally transformative, acting as a device utilised by people to make sense of their lives (Dill-Shackleford et al., 2016). We can see these observations borne out in the interview above as Riana utilises this TV show to discuss the ethical implications of cloning. She has been watching the show with her father who introduced her to it when she began studying genetics. Riana tells me that this sparks “big discussions” about genetics with him ranging from the acceptability of genetic testing to the ethics of who can own genetic information.

Another example of an in-depth use fiction is the aforementioned film *GATTACA*. Here Neville (Man aged 49) uses this film to initiate a discussion with his partner Vicki (Woman age 54) about the ethics of selection and genetics.

Vicki and Neville then move back and forth between real life scenarios, such as selective abortions in China and India, parental desire to take advantage of genetic testing and the fictional narrative presented in *GATTACA.*

**Vicki:** in many places in the world certainly here there are times when you're allowed to abort a child and times when you're not and there are places like India where they specifically say you know you're not allowed to choose to abort this child just because it's a girl but if you're capable of doing the tests that might suggest you know maybe this child might be more intelligent or maybe they will have this gene maybe they will be tall any therefore more beautiful umm or you know parents will do it even if it's illegal

**Neville:** well that's what happened in the film and anybody who doesn't do it has a faith child but it says anybody who doesn't match the genetic requirements are kept then or given menial jobs

This type of in-depth discussion about film narratives is rare in the data with only three interviews containing this type of discussion. Two interviews utilise dystopian fiction; *Orphan Black* and *GATTACA*. The final example is from an interview with Jane (37) and Dan (41) with their Children, Simon (12) and Gemma (7). Here the film utilised is *Jurassic World* (2015) the sequel to *Jurassic Park (1993)*. Films in the Jurassic Park franchise explore the tropes such as the dangers of playing with nature, the corruption of science by industry and the irresponsible scientist (Cormick, 2006). However, in this interview the film is used with irony and humour. Simon in particular enjoyed *Jurassic Park*. He described having watched all the films and played some of the video games. I ask if he has seen the latest film, which led to a family consensus that it wasn't as good as the original. Simon then moves on to describe the plot.

**Simon:** so they made a new dinosaur a hybrid out of DNA from a Velociraptor and T-Rex

**Interviewer:** OK

**Simon:** to make the Endominus-rex which was a very very clever yet very very dangerous dinosaur

**Interviewer:** yes

**Simon**: so it's nearly twice as long as a T-rex but it's got the brain of a Velciraptor so that's no great especially when it breaks out of it's enclosure

**Jane**: which it inevitable does (Laugher)

**Dan**: there was some plot line I mean I don't wish to spoil it for you (Laughter)

**Dan**: there was some plot line about a frog wasn't there was that what made it bad or was that its ultimate downfall

**Simon**: ohh um it would have been easier to tackle if Dr Wu hadn't put some weird stuff into it so basically to get the DNA the missing DNA they used frog DNA

**Jane**: What there was like a bit missing in the sequence was there

**Simon**: yeah and well they used this in all the dinosaurs

**Jane**: Ohh

**Simon**: and then for some strange reason Dr Wu also put cuttlefish DNA in it I'm not sure why

**Jane**: Isn't that about the colour changing thing

**Simon**: yes! That's why it could turn practically invisible and appear out of nowhere

**Dan**: I think Dr Woo really needs a visit from health and safety

The conversation moves on to Simon and his younger sister discussing which dinosaurs they should have mixed together to make a friendlier one than the ‘Endominus-rex’

**Simon**: Yeah they don't seem like two of the best dinosaurs I'd have perhaps got Dr Wu to get two of the softer ones maybe like a diplodocus and a

**Gemma:** triceratops

**Simon**: triceratops that would be cool

**Gemma**: diplodocus with three horns!

There are a number of things to note above. First is the humour and irony used when describing the film. For example, there is recognition of dramatic demands of the plot in Jane's light-hearted comment that the dinosaur will inevitably break out of its enclosure. This humour is also seen where Dan suggests that Dr Wu (the scientist who is responsible for creating new dinosaurs by combing different DNA) should get a visit from health and safety. Second is the way that the family members then go on to extemporise with the plot line, talking about what dinosaurs they would like to see Dr Wu create. This has similarities to the way Weinstein (2006) describes fan fiction as ‘playful engagements’ with science (p 618).

We can think of the way participants uses popular culture in this manner as examples of “habitable spaces” described my Michel de Certeau in *The Practice of Everyday Life* (1984). De Certeau describes the ways that people re-appropriate dominant representations by adapting them for their own interests. When people read, for example, they utilise the texts in new ways such as reading in a different order than intended, inferring meaning from small sections and reading their own memories and meanings into the text. De Certeau describes texts as *habitable*, furnished by the ideas and beliefs of the person consuming it. In this respect consumers are not passive by creatively engage with culture.

Jurassic Park here represents a “habitable space” furnished by the ideas and beliefs of the family. The discussion reflects the films story but also the family's interest in science - Simon tells me he wants to be a palaeontologist when he is older. Furthermore, the way the family engage with the Jurassic Park films allows them to explore their own family culture, in particular their sense of humour. We can see above they creatively engage with the text rather that consuming it uncritically. We can return here to the comment made by the president of the Federation of Australian Scientific and Technological Societies that “worrying science literacy levels demonstrates that students have perhaps been learning about science through Jurassic Park instead of through the education system” (quoted in Orthia et al., 2012, p.150). In the example above members of the family, especially 12-year-old Simon, do indeed demonstrate their familiarity with genetics by describing the techniques of genetic engineering in the Jurassic Park films. Additionally, they show an awareness of the ‘irresponsible scientists’ archetype in the form of Dr Wu. However, the manner in which the family jokes about the film and ‘plays’ with the plot demonstrates the creative ways this family utilise the film. The shared interest in *Jurassic Park* provides this family with opportunities to display their knowledge gained from the media and to create their own dialogue and narrative with the films.

In the sections above we have outlined some ways that participants were able to draw on popular culture to engage with genetics. Participants used references from popular culture as a cultural resource drawing on narratives, imagery and metaphors to talk about genetics in both discourses of hope and fear with some fictional narratives were used in more depth as ‘sense making devices’ allowing people to explore the moral and ethical implications of genetics.

## 8 Discussion and implications

Popular culture is often represented a low status or ‘everyday’ form of knowledge. While many people have enjoyable experiences with popular culture, this experience does not often transfer to formal or academic realms (Hall, 2012; Heron-Hruby and Alvermann, 2009). In education and science communication settings popular culture is often presented as a source of inaccurate information about science (Vackimes, 2010). The public often positioned as, at best, undiscriminating consumers and at worst victims of distorted scientific information.

The analysis presented in this study provides some evidence that popular culture, at least for our participants, within science fiction, documentaries, the news and celebrity, represents a valuable resource people are able to utilise when talking about genetics (and also science in general). In part, this is due to the rhetorical value of popular culture references. Popular culture contains a rich source of metaphors and narratives that participants used to frame and easily communicate their beliefs about genetics. The narratives found in science fiction have *cultural resonance* (Gamson and Modiglani, 1989) and provide a resource that allows participants to communicate their fears or anxieties about genetics and science in general.

This research also demonstrates that our participants are not passive consumers of media. For example, some were able to use narratives from popular culture to talk about the ethical implications of genetics while maintaining an awareness of fictional nature of these texts. Furthermore, popular culture became a ‘habitable space’ (De Certeau, 1984) as people adapted and furnished these references for their own interests and beliefs.

There are implications here for genetic counselling practice. In clinical genetic counselling practice, patients consistently rate higher satisfaction when counselling is emphasised over teaching (Austin et al., 2014). Furthermore, evidence suggests that educational goals of genetic counselling are improved by attending to the psychosocial needs of patients (Austin et al., 2014). Storytelling and narratives about science offer a window into a patient's emotional response (Trees et al., 2010). Encouraging patients to articulate their beliefs and feelings seems to facilitate their cognitive processing of complex medical information. Research also demonstrates that for patients to make meaning out of a genetic test, they must integrate the scientific information with their beliefs and values (Weil, 2000). Simply trying to impose genetic knowledge can lead to resistance as it deprives patients of valued beliefs, such as explanations of inheritance that help them make meaning of disease (McAllister, 2003). Effective communication in genetic counselling therefore is aided by listening and understanding the ways patients make sense of genetic information. As such, genetic counselling is enhanced when patients are free to articulate their beliefs, attitudes and opinions and when they are free to make sense of genetic on their own terms. Popular culture is an important way that people become familiar with genetics and can make sense of the ethical and moral aspects of genetic technologies. As such we argue that value could be added to the genetic counselling process is consideration is given to the broad range of patients' knowledge and experience, including their enjoyment of popular culture.

We do not suggest that popular culture can somehow replace scientific knowledge. Indeed, there are many cases where it is still important to provide accurate medical information. In the genetic counselling clinic, the provision of information remains a vital part of allowing patients to make informed, autonomous decision. However, this research provides evidence that there may be much to be gained by bringing patient's own interests -such as popular culture-into the clinic consultation. Even in ‘formal’ situations, such as a clinical consultation communication can be enriched by drawing on people's own knowledge and experience – described in educational theory as their ‘funds of knowledge’ (Moll, 2005). It is also possible that using references from popular culture can diminish power hierarchies creating what Lewis and Thomas (2017) call a “buffer zone” referring to the more neutral conversations that can happen when people from different backgrounds discuss science through art.

This study also provides further impetus for recognising that communication is rarely linear. A significant criticism levied at popular culture is that it contains frightening, inaccurate or deterministic messages (Dar-Nimrod and Heine, 2011) There is perhaps an irony here. Scholars and policy makers worried about genetics in popular culture have voiced concerns that genetics is represented too simplistically: genes are ascribed a power of causation that encourages genetic determinism. Yet in much of this research there is a similar implicit reductionism about the effects of popular culture; causation is presumed to be simple. As there is no ‘gene for’ complex traits; there is no ‘message for’ complex beliefs. This insight has broad implications for any science engagement activity surrounding genetics, such as genetic counselling. Any practitioner must recognise that their message will be actively interpreted. Clinic letters and conversations, blogs, talks science festivals etc all have the potential to become ‘habitable spaces’ furnished by the beliefs and knowledge of the publics and patients. This provides an opportunity for dialogue, and consequently for those engaged in science communication and genetics, this represents an opportunity for rich, genuine engagement.

## Grants and funding

Jonathan Roberts and Anna Middleton are supported by Wellcome [206194] funding to the Society and Ethics Research Group, and Public Engagement Group, Connecting Science, Wellcome Genome Campus. Jonathan Roberts is also funded by the Rosalind Driver Scholarship fund (King's College London).

## Appendix A Supplementary data

Data Profile Data Profile
